# JNK1 Protects against Glucolipotoxicity-Mediated Beta-Cell Apoptosis

**DOI:** 10.1371/journal.pone.0087067

**Published:** 2014-01-24

**Authors:** Michala Prause, Dan Ploug Christensen, Nils Billestrup, Thomas Mandrup-Poulsen

**Affiliations:** 1 Endocrinology Research Section, Department of Biomedical Sciences, University of Copenhagen, Copenhagen, Denmark; 2 Section of Cellular and Metabolic Research, Department of Biomedical Sciences, University of Copenhagen, Copenhagen, Denmark; 3 Department of Molecular Medicine and Surgery, Karolinska Institute, Stockholm, Sweden; University of Lille Nord de France, France

## Abstract

Pancreatic β-cell dysfunction is central to type 2 diabetes pathogenesis. Prolonged elevated levels of circulating free-fatty acids and hyperglycemia, also termed glucolipotoxicity, mediate β-cell dysfunction and apoptosis associated with increased c-Jun N-terminal Kinase (JNK) activity. Endoplasmic reticulum (ER) and oxidative stress are elicited by palmitate and high glucose concentrations further potentiating JNK activity. Our aim was to determine the role of the JNK subtypes JNK1, JNK2 and JNK3 in palmitate and high glucose-induced β-cell apoptosis. We established insulin-producing INS1 cell lines stably expressing JNK subtype specific shRNAs to understand the differential roles of the individual JNK isoforms. JNK activity was increased after 3 h of palmitate and high glucose exposure associated with increased expression of ER and mitochondrial stress markers. JNK1 shRNA expressing INS1 cells showed increased apoptosis and cleaved caspase 9 and 3 compared to non-sense shRNA expressing control INS1 cells when exposed to palmitate and high glucose associated with increased CHOP expression, ROS formation and *Puma* mRNA expression. JNK2 shRNA expressing INS1 cells did not affect palmitate and high glucose induced apoptosis or ER stress markers, but increased *Puma* mRNA expression compared to non-sense shRNA expressing INS1 cells. Finally, JNK3 shRNA expressing INS1 cells did not induce apoptosis compared to non-sense shRNA expressing INS1 cells when exposed to palmitate and high glucose but showed increased caspase 9 and 3 cleavage associated with increased *DP5* and *Puma* mRNA expression. These data suggest that JNK1 protects against palmitate and high glucose-induced β-cell apoptosis associated with reduced ER and mitochondrial stress.

## Introduction

The incidence of obesity and Type 2 diabetes is increasing worldwide as a consequence of sedentary lifestyle and excess caloric intake, in particular saturated fats and simple carbohydrates [1]. Obese and diabetic subjects have elevated plasma levels of nonesterified fatty acids (NEFAs) and hyperglycemia, which are believed to cause decreased insulin synthesis and impaired glucose responsiveness in pancreatic β-cells, also termed glucolipotoxicity [2], [3]. Chronic exposure of β-cells to high NEFAs and glucose concentrations results in β-cell dysfunction and loss by ER stress and oxidative stress [4]–[6] resulting in apoptosis [4], [7]–[9].

The ER stress response, also known as the unfolded protein response (UPR), is a complex signaling network initiated to restore normal ER homeostasis by decreasing protein load and increasing protein folding capacity. Upon ER stress, UPR is initiated by dissociation of the ER chaperone immunoglobulin heavy chain binding protein (Bip) from the ER membrane resident proteins; eukaryotic translational initiation factor-2α kinase 3 (PERK), inositol-requiring enzyme 1 (IRE1) and activating transcription factor 6 (ATF6) thereby activating these proteins. Activated PERK phosphorylates and inhibits eukaryotic initiation factor 2 (eIF2) leading to global translational attenuation. However, certain mRNAs gain a selective advantage for translation under these conditions e.g. activating transcription factor (ATF4). ATF4 activates the transcription of C/EBP homologous protein (CHOP), thought to mediate palmitate-induced β-cell death [10], [11]. Active IRE1 splices X-box binding protein-1 (Xbp)-1 mRNA, translating into an active transcription factor sXbp-1 that induces ER chaperones and ER-associated protein degradation. Activated ATF6 also mediates transcription of genes encoding ER chaperone proteins. Detection of increased ER stress marker expression including ATF3, Bip and CHOP in mouse islets exposed to elevated lipids and high glucose and in β-cells of type 2 diabetic patients supports the involvement of ER stress in the pathogenesis of Type 2 diabetes [12]–[14].

Prolonged and excessive ER stress induced β-cell apoptosis is associated with c-jun N-terminal kinase (JNK) activation [9], [15]. JNK comprises a family of three JNK subtypes, JNK1, JNK2 and JNK3, and the three JNK genes; *jnk1*, *jnk2* and *jnk3* encode more than 10 different isoforms [16], [17]. Despite high JNK isoform homology the JNK subtypes have differential functions depending of cellular context and stimuli [18], [19]. In proinflammatory cytokine-induced β-cell apoptosis JNK activation is very rapid and transient [20]. However, lipo- and glucolipotoxicity-induced ER stress dependent β-cell apoptosis is characterized by a late and more prolonged JNK activation, and blocking JNK activity with the JNK inhibitory small molecule SP600123 *in vitro* decreases lipotoxic- and glucolipotoxic β-cell apoptosis [9], [21]–[24]. Additionally, JNK activity is potentiated by glucolipotoxicity via oxidative stress and mitochondrial ROS formation [4], [6], [25], [26]. ER stress cross-talks to the mitochondrial or intrinsic death pathway via p53-upregulated modulator of apoptosis (Puma) and JNK-dependent upregulation of the Death protein (DP5) [27].

However, the individual roles of the three different JNK subtypes in β-cell glucolipotoxicity are not clarified. We hypothesized that the JNK subtypes relay differentiated and balanced signaling in the β-cell response to glucolipotoxic stress. We therefore phenotyped INS-1 cells stably expressing JNK1, JNK2 or JNK3 shRNAs. We established glucolipotoxicity readouts, i.e. ER stress, ROS formation and JNK activity in INS-1 cells. We report that JNK1 shRNA aggravated palmitate and high glucose-induced toxicity associated with changes in ROS, CHOP and *Puma* expression, and conclude that JNK1 serves an antiapoptotic role in the β-cell response to glucolipotoxic stress.

## Materials and Methods

### Cell Culture and Reagents

The clonal rat β-cell line INS1 [28] kindly provided by C. Wollheim (Geneva, Switzerland) and INS1 cell lines stably expressing shRNA were grown in RPMI-1640 medium with 11 mmol/L glucose (RPMI-1640 with glutaMAX supplemented with 50 µmol/L β-mercaptoethanol, 100 Units/mL pencillin,100 µg/mL streptomycin and 10% heat-inactivated fetal bovine serum (FBS) (Life Technologies, Naerum, Denmark). Cells were incubated in a humidified atmosphere of 5% CO_2_ at 37°C. For experimental procedures culture medium with 1% FBS and 1% BSA was used. Palmitate, D-glucose and fatty acid-free low-endotoxin BSA were purchased from Sigma (Schnelldorf, Germany). Palmitate was solubilizied in 90% ethanol, heated to 60°C and used 1∶100 dilution in media.

JNK1 antibody (F-3) (sc-1648) was from Santa Cruz technologies (Santa Cruz, CA, USA). JNK-2 (#4672), JNK-3 (#55A8), cleaved caspase 3 (D175) (#9661), cleaved caspase-9 (Asp353) (#9507) and p-eIF2α (Ser51) (#3597) antibodies were from Cell Signalling (Beverly, MA, USA). GADD153 (CHOP) and β-actin (ab6276) antibodies were from Abcam (Cambridge, UK). The specificity of the JNK antibodies were verified against recombinant JNK1, JNK2 and JNK3 protein Abnova (Tappei, Taiwan). SP600125 (#S5567) was from Sigma.

HEK293FT cells used for Lentivirus production were cultured in D-MEM medium (D-MEM high glucose, glutaMAX, Life Technologies), supplemented with 100 Units/mL pencillin, 10 nM MEM non-essential amino acids, 1 mM sodium pyruvate and 10% certified FBS (Life Technologies).

HT1080 cells used for determining the virus titer were cultured in D-MEM medium (D-MEM high glucose, glutaMAX, Life Technologies) supplemented with 100 Units/mL pencillin and 100 µg/mL streptomycin and 10% certified FBS (Life Technologies).

### Lentivirus

Lentiviral vectors pLKO.1 JNK1 (TRCN0000055115), pLKO.1 JNK2 (TRCN0000012590), pLKO.1 JNK3 (TRCN0000012634) and empty vector were all from Open Biosystems, Thermo Scientific (St. Leon-Rot, Germany). pLKO.1 non-sense (SHC002) was from Sigma. All constructs contained a puromycin-resistance gene as mammalian selection marker.

HEK293FT cell medium was changed to OPTI-MEM medium (Life Technologies) without antibiotics 8 h prior to transfection. pMD2.G envelope plasmid, psPAX2 packing plasmid and pLKO.1 shRNA plasmid from the Trono Lab (Lausanne, Switzerland) were mixed in a ratio of 1∶3∶4 respectively in OPTI-MEM. Lipofectamine 2000 (Life Technologies) was diluted with OPTI-MEM without serum. After 5 min incubation the diluted Lipofectamine 2000 and DNA were mixed gently and incubated for 20 min at room temperature. Next the DNA-Lipofectamine 2000 complexes were added to the 293FT cells and incubated for 15–17 h. The next day transfection medium was removed and replaced with 10 ml D-MEM complete medium without antibiotics. 72 h post transfection medium containing virus was collected and centrifuged at 1500 rpm at 20°C to remove cell debris before filtering through a Millex-HV 0.45 µm filter and purified using ultracentrifugation at 20,000 rpm for 2 h. The pellets containing virus were dissolved in INS1 culture medium and stored at −80°C.

A titration curve was performed in a 10-fold serial dilution of each Lentiviral supernatant (10^2^–10^6^ dilutions) or with supernatant from untransduced INS-1 cells (mock) to determine the concentration of the virus produced. HT1080 cells were infected with Lentivirus, and stably transduced cells were screened using 1 µg/ml puromycin. Next we stained for the number of pyromycin-resistant colonies using a 1% crystal violet solution in 10% ethanol. Multiplicity of infection, MOI, was determined in INS1 cells infected with 5, 10 or 20 Lentivirus-particles per cell for 8 h. 48 h post-infection protein was harvested and JNK1, JNK2 and JNK3 knockdown verified by Western blot analysis. INS1cell lines stably expressing JNK1, 2 or 3 shRNAs, nonsense shRNA or empty vector were created by infecting cells with an MOI of 5 and selecting stably transduced cells using 1 µg/ml pyromycin. Cell lines were passaged a minimum of three times and tested for knockdown efficiency and specificity compared to empty vector and nonsense shRNA expressing cell lines.

### Insulin Content

0.05×10^6^ INS1 cells stably expressing JNK1, JNK2, JNK3 or non-sense shRNA were cultured 48 h prior exposure to 0.5 mM palmitate and 25mM glucose or vehicle for 24 h. After pre-culturing for 2 h in KREBS Ringer (KR) containing 1M HEPES, 2 mM glutamine, 5 mM NaHCO3, 0.2% BSA and adjusted to pH 7.4, the cells were challenged with 2 mM glucose in KR for 1 h, medium was removed and 20 mM in KR was added to the cells for 1 h. Next the cells were given 20 mM in KR supplemented with 40 µM forskolin for 1 h. Cells were lysed (sonication: 40 W for 2×15 s) and saved for determination of insulin and DNA content measured by Quant-iT Picogreen dsDNA (Life technologies) in accordance to the manufacturer’s protocol. Total insulin content was measured by competitive ELISA as described in [29].

### cDNA and qRT-PCR

INS1 cells stably expressing JNK1, JNK2, JNK3 or non-sense shRNA were cultured 24 h prior to exposure to 0.5 mM palmitate and 25 mM glucose or vehicle for 4–24 h, and total RNA was isolated using the Nucleospin RNAII kit (Macherey-Nagel, Düren, Germany) and quantified on the NanoDrop 1000 micro-volume spectrophotometer. cDNA was synthesized from 500 ng of RNA using the iScript Reverse Transcription kit (Biorad, Copenhagen, Denmark) following the manufacturer’s protocol. Quantitative RT-PCR was performed in a 10 µl volume using 1.2 µl of mixed primers (final concentration 600 nM), 5.0 µl SYBR green Master Mix (Applied Biosystems, Carlsbad, CA, USA), 3.3 µl H_2_O and 0.5 µl 25 ng template and run on a 384-well plate in triplicate on the Applied Biosystems Prism 7900HT real-time PCR machine for 40 cycles. Each cDNA sample was subjected to two individual PCR amplifications either for the gene of interest of for the reference gene *Hprt1*. The results were analyzed using SDS 2.3 software (Applied Biosystems). The primer sequences are listed in Table 1 and synthesized by TAG Copenhagen, Copenhagen, Denmark.

**Table 1:** Primer sequence.

Target	Forward	Reverse
*Hprt1*	GCAGACTTTGCTTTCCTT	CCGCTGTCTTTTAGGCTT
*Xbp1s*	GAGTCCGCAGCAGGTG	GCGTCAGAATCCATGGGA
*CHOP*	CAGCGACAGAGCCAAAATAAC	TGTGGTGGTGTATGAAGATGC
*ATF3*	GGAGTCAGTCACCATCAACAA	CGCCTCCTTTTTCTCTCATCT
*ATF4*	GTTGGTCAGTGCCTCAGACA	CATTCGAAACAGAGCATCGA
*DP5*	GCCGTGGTGTTACTTGGA	GATTGTGCCAGAGCTTCACA
*Puma*	AGTGCGCCTTCACTTTGG	CAGGAGGCTAGTGGTCAGGT
*Ins1*	GTCCTCTGGGAGCCCAAG	ACAGAGCCTCCACCAGG
*Ins2*	ATCCTCTGGGAGCCCCGC	AGAGAGCTTCCACCAAG

### Western Blot Analysis

2×10^6^ INS1 cells stably expressing JNK1, JNK2, JNK3 or non-sense shRNA were cultured 24 h prior to exposure to the indicated concentrations of glucose, palmitate or vehicle for 16–24 h, were washed in ice-cold PBS and lysed for 15 min on ice. The protein concentration was measured by the Bradford method (Bio-Rad). Protein was separated by SDS-PAGE and transferred onto PVDF membranes (Expedeon, Cambridgeshire, UK). Blots were blocked for 1 h in 5% BSA and afterwards incubated overnight at 4°C with primary antibody. Blots were washed and incubated with a rabbit secondary horseradish peroxidase-conjugated antibody for 1h. Immune complexes were detected by chemiluminescence using Supersignal West Dura Extended Duration Substrate (Thermo Scientific), and images were captured digitally by use of the Alpha Innotech FlourChem Q imaging platform (Kemen Tec, Taastrup, Denmark).

### Cell Death Detection Assay

Apoptotic cell death was measured by the detection of DNA-histone complexes released from the nucleus to the cytosol of cells using Cell Death Detection ELISA^PLUS^ (Roche, Hvidovre, Denmark) as described by the manufacturer. In brief, 0.05×10^6^ INS1 cells stably expressing JNK1, JNK2, JNK3 or non-sense shRNA were cultured 24 h prior to exposure to 0.5 mM palmitate and 25 mM glucose or vehicle. After an additional 24 h the culture medium was removed and cells lysed in 200 µL 1× lysis buffer for 30 min at room temperature. The lysate was then centrifuged for 10 min at 200×g, incubated with anti-DNA peroxidase and anti-histone-biotin and added to streptavidin-coated wells for 2 h at room temperature. Absorbance was measured after addition of peroxidase substrate ABTS (2.2-azini-bis-3-ethylbenzthiazoline-6-sufonate) at 405 and 490 nm.

### Detection of Reactive Oxygen Species

0.05×10^6^ INS1 cells stably expressing JNK1, JNK2, JNK3 or non-sense shRNA were seeded in clear-bottom black 96-well plates and pre-incubated for 48 h prior to 24 h of culture in the presence of 25 mM glucose and 0.5 mM palmitate. Cells were then washed twice with KR containing 5mM HEPES, 5 mM glucose and 0.15% NaHCO_3_ and adjusted to pH 7.4. Production of reactive oxygen species (ROS) was assessed by fluorescent detection using a final concentration of 10 µM CM-H2DCFDA (#C6827) (Life Technologies) in KR. Fluorescence was measured according to the manufacturer’s instruction at time points 0 (reference) and 45 min following addition of CM-H2DCFDA. Data presented are fluorescent signals after 45 min subtracted the reference.

### Statistical Analysis

The data are shown as means ± SEM and were analyzed using GraphPad Prism (GraphPad Software Inc., San Diego, CA, USA) and SAS (SAS Institute version 9.2, Cary, NC, USA). Statistical significance was determined using one or two-way ANOVA with Bonferroni- or Dunnet-corrected *post hoc* tests as appropriate. P-values less than or equal to 0.05 were considered statistically significant.

## Results

### JNK1 Knockdown Increases Palmitate and Glucose-induced β-cell Apoptosis

**Figure 1:**
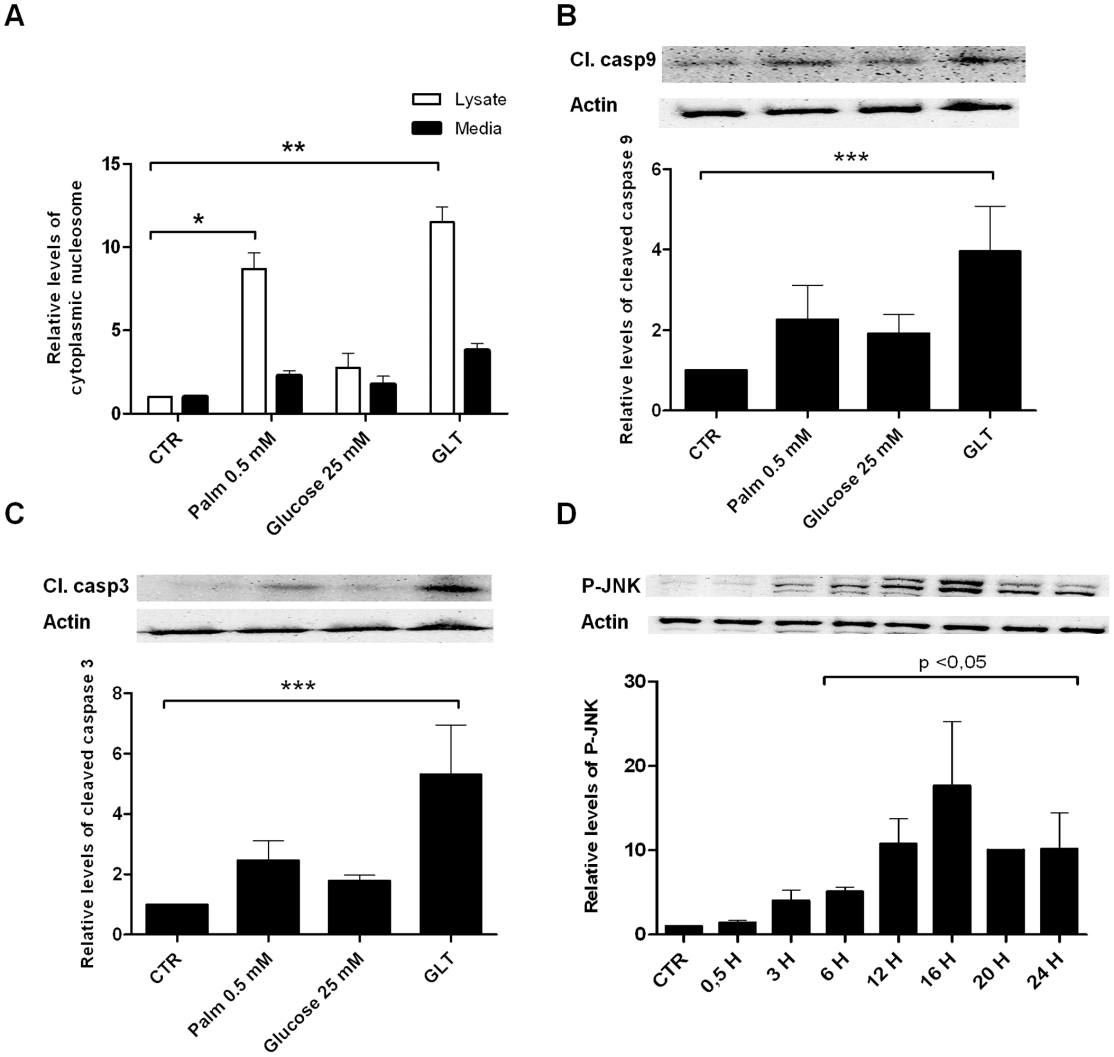
Palmitate and high glucose induce apoptosis, cleaved caspase 9, 3 and JNK phosphorylation in INS1 cells. INS1 cells were exposed to 0.5(GLT) or vehicle (CTR) for 24 h. A: Apoptosis was measured as the relative levels of cytoplasmic nucleosomes compared to CTR in INS1 cell lysates or in exposure medium using the Roche Cell Death detection ELISA kit. Data are shown as means+SEM of four independent experiments. B, C: Cleaved caspase 9 or 3 was assessed by Western blotting and normalized to actin. Data are shown as means+SEM of four independent experiments; representative blots are shown. D: Time-course analysis of P-JNK protein expression analyzed by Western blotting. Protein was isolated from INS1 cells exposed to 0.5 mM palmitate and 25 mM glucose for 0.5–24 h. Actin was used as loading control; representative blots are shown. Data are shown as means+SEM of three independent experiments. *P<0.05, **P<0.01, ***P<0.001.

In line with previous reports, exposure of INS1 cells to 0.5 mM palmitate for 24 h significantly induced apoptosis in INS1 cells and caused approximately 2-fold increase in cleaved caspase 9 and 3 (fig. 1A, B, C). Addition of 25 mM glucose to 0.5 mM palmitate aggravated apoptosis and caspase 9 and 3 cleavage. Palmitate and high glucose increased phosphorylation of JNK protein from 3–24 h with peak activity at 16 h as evidence of increased JNK kinase activity (fig. 1D).

**Figure 2:**
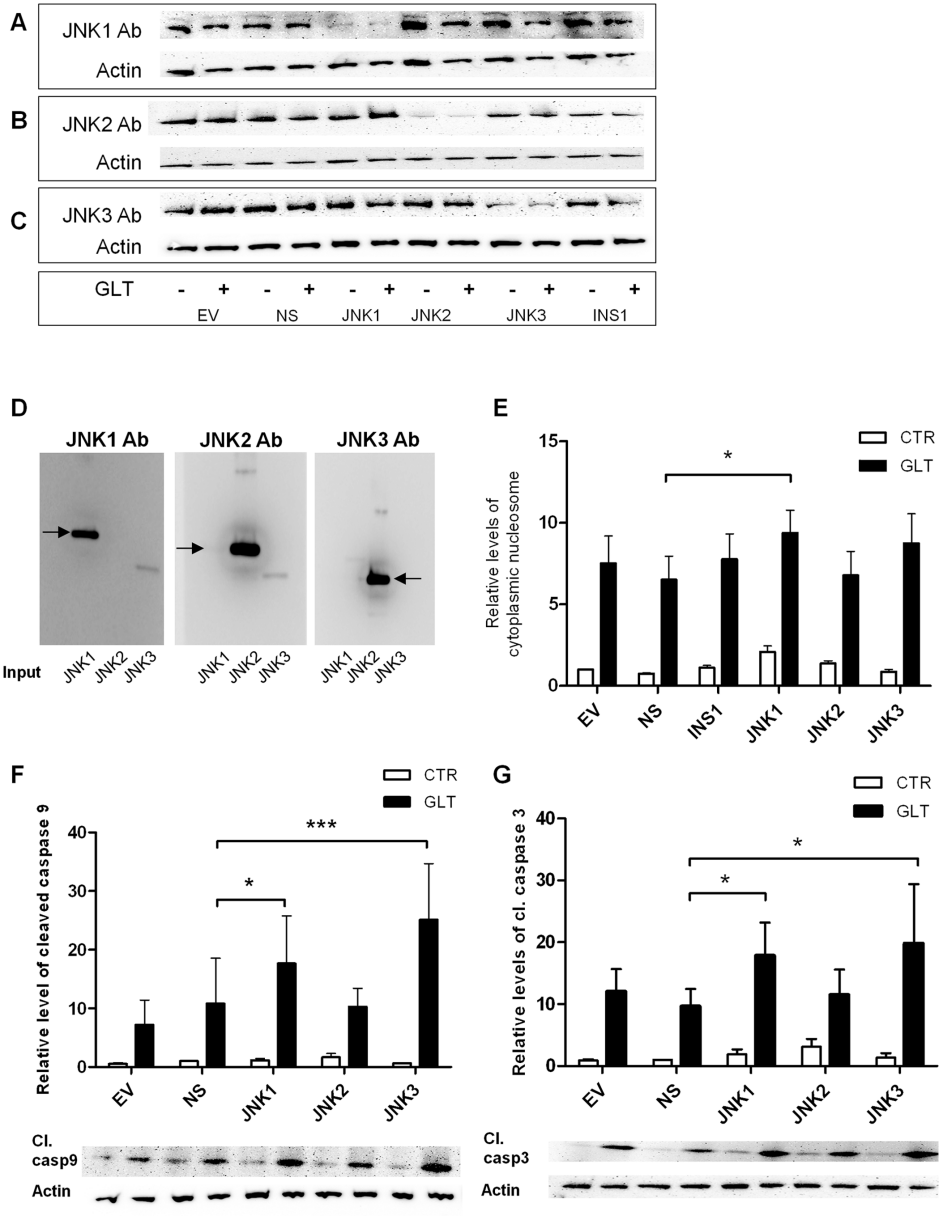
Figure 2. JNK1 knockdown increases palmitate and high glucose-induced β-cell apoptosis. INS1 cell lines stably expressing shRNA for JNK1, JNK2, JNK3, non-sense shRNA (ns), empty vector controls (ev) or wildtype INS1 cells were exposed to 0.5 mM palmitate and 25 mM glucose (GLT) (+) or vehicle (−) for 24 h. A: JNK1, B: JNK2, C: JNK3 knockdown specificity and efficiency in JNK1, 2 and 3 shRNA expressing INS1 cell lines were assessed by Western blotting with actin as loading control. Blots are representative of knockdown efficiency in the shRNA expressing INS1 cell lines. D: The specificity of the JNK antibodies were verified against JNK1 (73 kDa with GST tag), JNK2 (72 kDa with GST tag) and JNK3 (61 kDa with GST tag) recombinant proteins. INS1 cell lines stably expressing shRNA for JNK1, JNK2, JNK3, non-sense shRNA (ns), empty vector controls (ev) or wildtype INS1 cells were exposed to 0.5 mM palmitate and 25 mM glucose (black bars) or vehicle (white bars) for 24 h. E: Apoptosis was measured as the relative levels of cytoplasmic nucleosomes in INS1 shRNA stable cell lines lysates compared to ns vehicle using the Roche Cell Death detection ELISA kit. Data are shown as means+SEM of five independent experiments. F, G: Cleaved caspase 9 or 3 was assessed by Western blotting and normalized to actin. Data are shown as means+SEM of five independent experiments; representative blots are shown. *P<0.05, ***P<0.001.

To examine the differential roles of the JNK1, 2 and 3 in palmitate and high glucose-induced apoptosis we created INS1 cell lines stably expressing shRNAs directed against JNK1, JNK2 or JNK3, a non-sense shRNA and an empty vector control. JNK1, 2 or 3 knockdown efficiency and specificity was confirmed by Western blotting (fig. 2A, B, C). The specificity of JNK1, 2 and 3 antibodies were verified using recombinant protein (fig. 2D). Palmitate and high glucose-induced apoptosis and caspase 9 and 3 cleavage after 24 h in wildtype INS1 cells, non-sense shRNA and empty vector expressing INS1 cells were determined (fig. 2E, F, G). Surprisingly, JNK1 knockdown increased palmitate and high glucose-induced apoptosis measured as an increased level of cytoplasmic nucleosomes (fig. 2E). JNK1, but also JNK3 knockdown, were accompanied by significantly increased levels of cleaved caspase 9 and 3, while JNK2 knockdown affected neither palmitate and high glucose-induced apoptosis nor caspase 9 and 3 cleavage following palmitate and high glucose exposure (fig. 2F, G).

### JNK Knockdown does not Affect Palmitate and High Glucose-induced Expression of the ER Stress Markers *sXbp-1*, p-eIF2α, *ATF4* or *ATF3*

**Figure 3:**
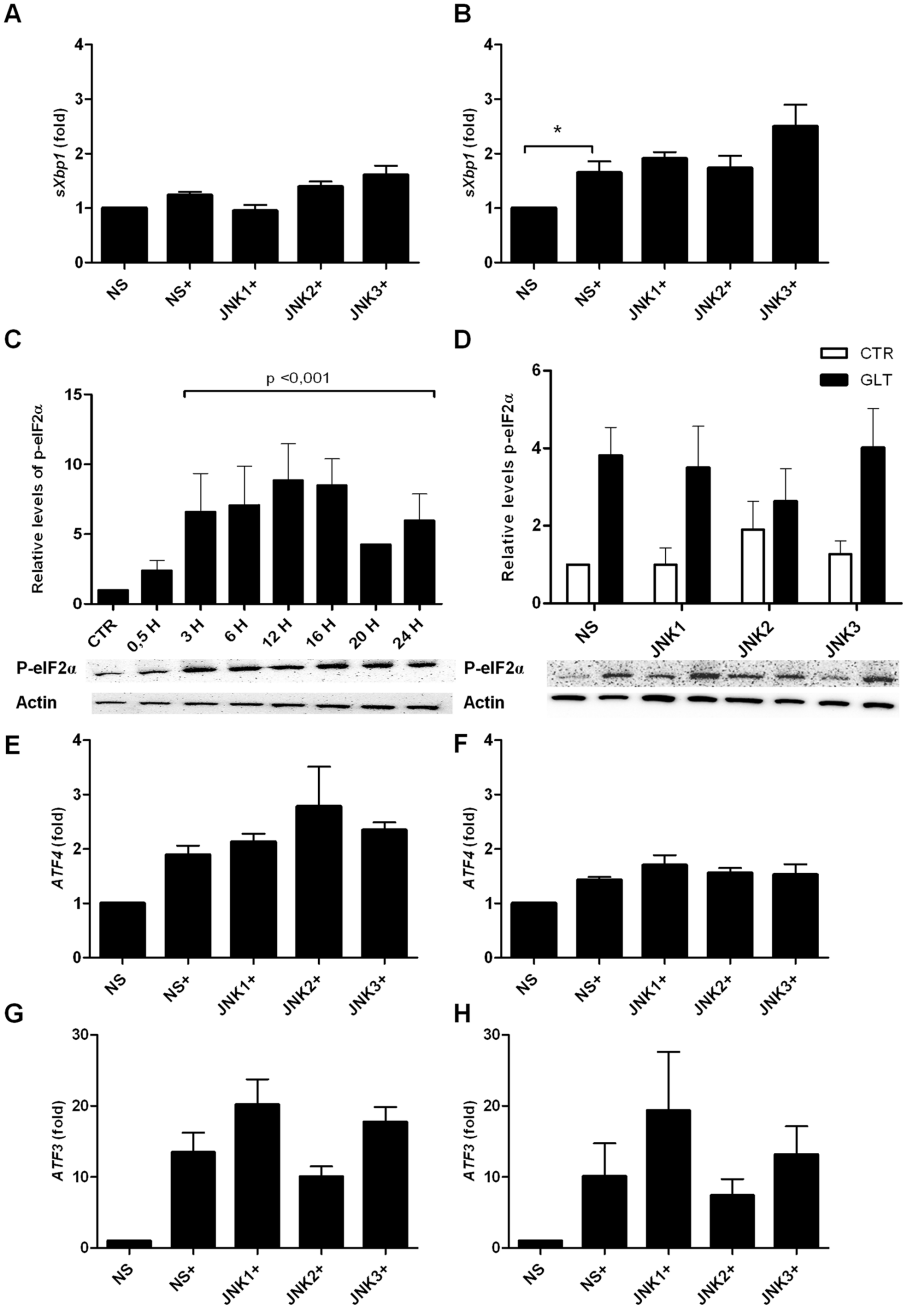
JNK knockdown does not affect *sXbp-1*, P-eIF2α, *ATF4* and *ATF3* expression. INS1 cell lines stably expressing shRNA directed against JNK1, JNK2, JNK3 or the non-sense (ns) control were exposed to 0.5 mM palmitate and 25 mM glucose for 4–16 h (+). Relative mRNA expression was measured using quantitative RT-PCR and normalized to *Hprt1*. Relative *sXBP1*mRNA expression at A: 4 h, B: 12 h. C: Time-course analysis of P-eIF2α protein expression analyzed by Western blotting. Protein was isolated from INS1 cells exposed to 0.5 mM palmitate and 25 mM glucose for 0.5–24 h. Actin was used as loading control, representative blots are shown. Data are shown with+SEM of three independent experiments. D: Protein was isolated from INS1 cell lines stably expressing JNK1, JNK2 or JNK3 shRNA or the non-sense (ns) shRNA control after 16 h of palmitate and high glucose exposure, and p-eIF2α levels were analyzed by Western blotting. Actin was used as the loading control, representative blots are shown. Relative *ATF-4* mRNA expression at E: 12 h, F: 16 h. Relative *ATF-3* mRNA expression at G: 12 h, H: 16 h. Data are shown with+SEM of five independent experiments. *P<0.05, **P<0.01, ***P<0.001.

We next investigated if the increased apoptosis in JNK1 shRNA expressing INS1 cells exposed to palmitate and high glucose were mediated through modulation of proteins in the UPR. Blockage of JNK activity by SP600125 decreases *sXbp-1* mRNA in hypothalamic neurons exposed to palmitate, indicating an upstream role of JNK in ER stress [30]. *sXbp-1* mRNA expression was significantly upregulated in control INS1 cells expressing non-sense shRNA when exposed to palmitate and high glucose for 12 h but not 4 h (fig. 3A–B). However, JNK1, 2 and 3 knockdown did not affect *sXbp-1* mRNA levels (fig. 3A–B).

We therefore examined the PERK-pathway. In a time course study, palmitate and high glucose significantly increased eIF2α phosphoryation from 3–24 h (fig. 3C), indicating activation of the PERK-pathway upon glucolipotoxicity. Knockdown of JNK1, 2 or 3 did not affect the phosphorylation of eIF2α compared to non-sense shRNA expressing INS1 cells after 16 h of palmitate and high glucose exposure (fig. 3D). ATF4, a downstream transcription factor of eIF2α, was not regulated at the mRNA expression level in JNK1, 2 and 3 shRNA vs. non-sense shRNA expressing INS1 cells exposed to palmitate and high glucose for 12 or 16 h (fig. 3E–F) as was true for expression of *ATF3* (fig. 3G–H).

### JNK1 Knockdown Increases CHOP Expression and ROS Formation

**Figure 4:**
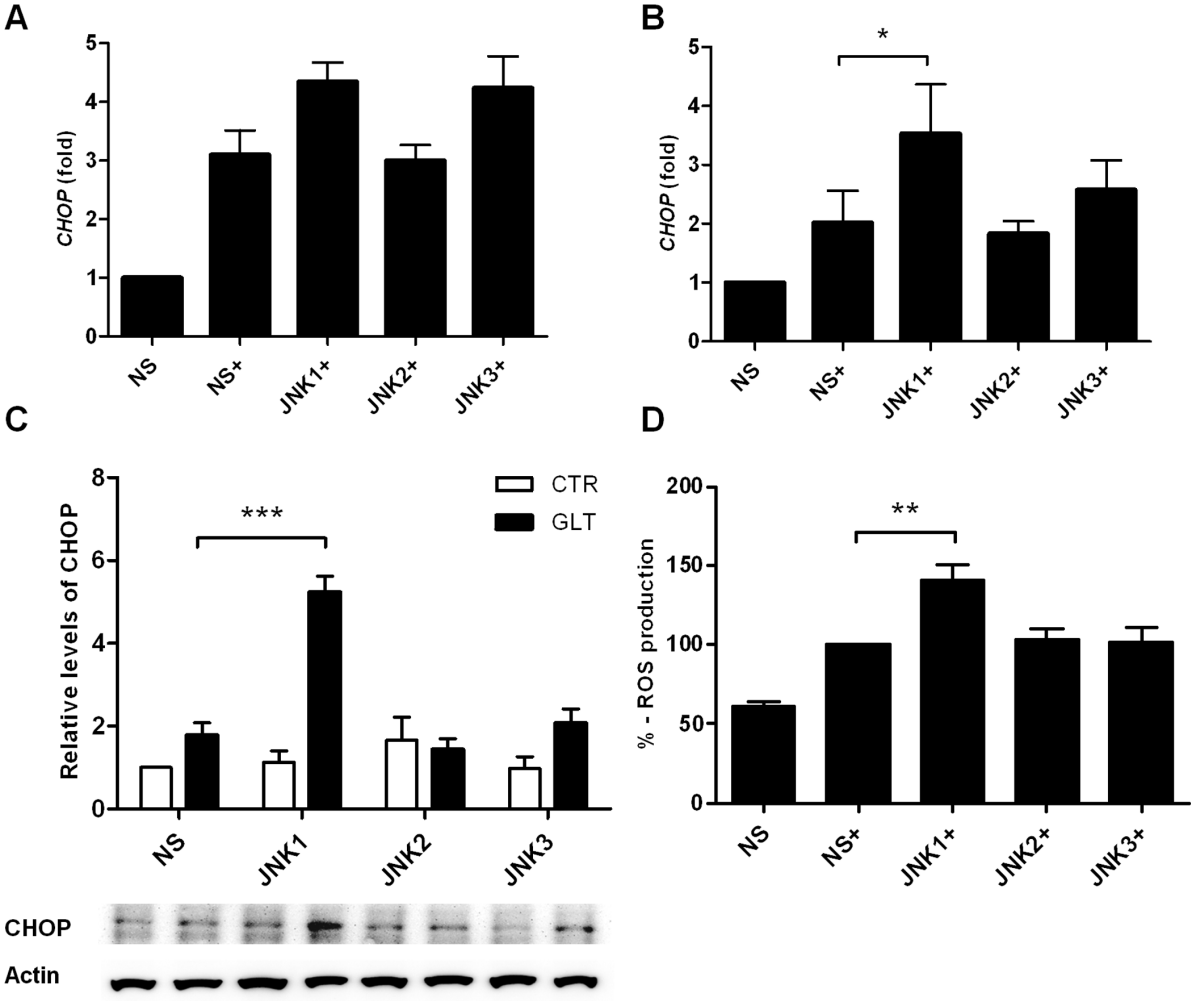
JNK1 knockdown increases CHOP expression and ROS formation. INS1 cells stably expressing shRNA directed against JNK1, JNK2, JNK3 or the non-sense (ns) control were exposed to 0.5 mM palmitate and 25 mM glucose for 12–24 h (+). Relative mRNA expression was measured using quantitative RT-PCR and normalized to *Hprt1*. Relative *CHOP* mRNA expression at A: 12 h, B: 16 h. C: Protein was isolated from JNK1, 2 or 3 shRNA expressing INS1 cells after 24 h of 0.5 mM palmitate and 25 mM glucose exposure. CHOP levels were analyzed by Western blotting. Actin was used as the loading control, representative blots are shown. Data are shown with ± SEM of five - six independent experiments D: ROS production was measured by a fluorophore probe as described in the methods section. JNK1, JNK2, JNK3 or non-sense (ns) shRNA expressing INS1 cells were exposed to palmitate and high glucose for 24 h and fluorescent signal was normalized to ns (100%). Data are shown+SEM of four independent experiments. *P<0.05, **P<0.01, ***P<0.001.

We next turned to investigate the importance of JNK subtypes for CHOP expression in palmitate and high glucose exposed INS1 cells. *CHOP* mRNA was upregulated by palmitate and high glucose at 12 and 16 h in non-sense shRNA expressing INS1 cells (fig. 4A–B). Only JNK1 knockdown significantly increased *CHOP* mRNA expression at 16 h (fig. 4B) and protein expression at 24 h (fig. 4C). CHOP deletion improves ER function and protects against oxidative stress in response to ER stress in β-cells [10]. We therefore investigated if increased CHOP expression in JNK1 shRNA expressing INS1 cells also resulted in increased ROS formation. INS1 cells stably expressing non-sense shRNA or JNK1, 2 or 3 shRNA were exposed to palmitate and high glucose for 24 h and examined for ROS production. Interestingly, knockdown of JNK1 significantly increased ROS production compared to non-sense shRNA, JNK2 and JNK3 shRNA expressing INS1 cells (fig. 4D).

### JNK1 Knockdown Modulates *Puma* Expression

**Figure 5:**
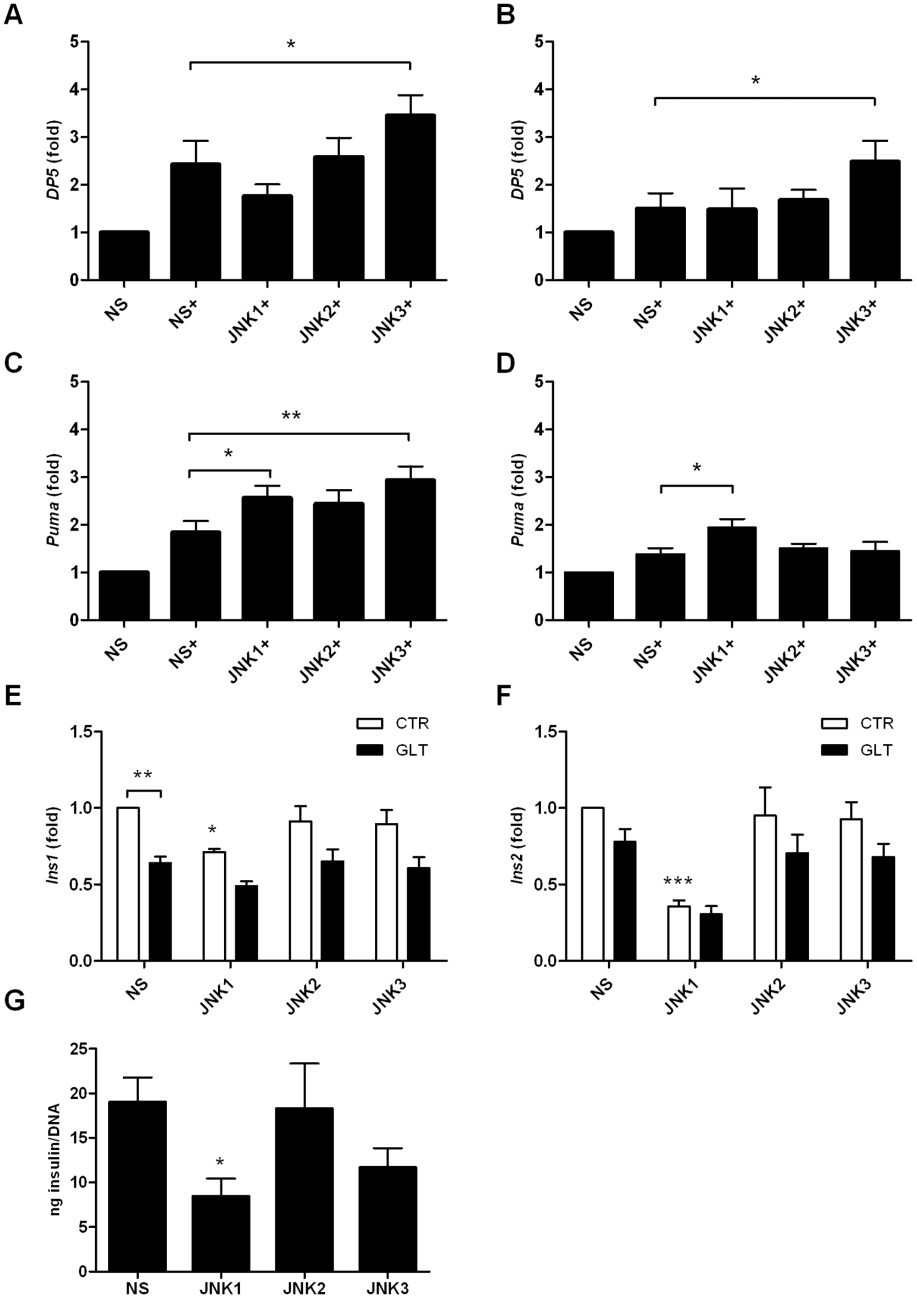
JNK knockdown increase *Puma* mRNA expression. INS1 cells stably expressing shRNA directed against JNK1, JNK2, JNK3 or the non-sense control were exposed to 0.5 mM palmitate and 25 mM glucose for 12 or16 h (+). Relative mRNA expression was measured using quantitative RT-PCR and normalized to *Hprt1*. Relative *DP5* mRNA expression at A: 12 h, B: 16 h. Relative *Puma* mRNA expression C: 12 h, D: 16 h. Data are shown with+SEM of five - six independent experiments. INS1 cells stably expressing shRNA directed against JNK1, JNK2, JNK3 or the non-sense control were exposed to 0.5 mM palmitate and 25 mM glucose (black bars) or vehicle (white bars) for 24 h. Relative *Ins1* mRNA expression E: 24 h, Relative *Ins2* mRNA expression, F: 24 h. G: Total insulin content (ng insulin/total DNA) after GSIS. Data are shown with+SEM of four independent experiments *P<0.05, **P<0.01, ***P<0.001.

As knockdown of JNK1 potentiated palmitate and high glucose-induced apoptosis in the INS1 cell line, resulting in increased levels of cleaved caspase 9 and 3 and ROS formation, we reasoned that JNK1 regulated the mitochondrial intrinsic death pathway. Inhibiting total JNK activity by SP600125 blocked expression of *DP5* known to contribute to palmitate-induced apoptosis through the mitochondrial death pathway in INS1E cells [27]. We therefore investigated if the β-cell protective effect of JNK1 against glucolipotoxicity was mediated through modulation of the BH3 only proteins DP5 and Puma, previously shown to be upregulated by and play a role in cytokine- and palmitate-induced β-cell apoptosis [27], [31], [32]. Interestingly, only JNK3 knockdown increased *DP5* mRNA expression compared to non-sense shRNA expressing INS cells at 12 and 16 h (fig. 5A–B). However, JNK1, 2 or 3 knockdown increased *Puma* mRNA expression after 12 h of palmitate and high glucose exposure (fig. 5C). Additionally, JNK1 knockdown significantly potentiated *Puma* mRNA expression at 16 h of glucolipotoxicity (fig. 5D).

To investigate the functional role of JNK knockdown in INS1 cells we measured the importance of JNK1, 2 and 3 knockdown at insulin gene transcription and content. JNK1 knockdown significantly reduced basal *Ins1* and *Ins2* mRNA expression (fig. 5E–F) and basal insulin content (fig. 5G) compared to non-sense shRNA expressing INS1 cells.

## Discussion

Here we report that stable knockdown of JNK1 by RNA interference in INS1 cells aggravated palmitate and high glucose-induced INS1 apoptosis, caspase 9 and 3 cleavage (fig. 2E–G). Knockdown of JNK3 induced caspase 9 and 3 cleavage but did not affect apoptosis. How the JNK proteins are activated by palmitate and high glucose is unclear, but it is suggested that palmitate initiates ER stress which activates JNK in turn exerting positive feedback on ER stress activation [24], [33]. It is debated if the Toll-like receptors2 and 4 (TLR2/4) are directly activated by palmitate, but TLR4 possibly contributes to JNK activation by binding free palmitate [22], [34]. The time-course of JNK phosphorylation in INS1 cells in response to palmitate and high glucose exposure showed JNK phosphorylation after 3 h, peaking at 16 h exposure (fig. 1D). JNK phosphorylation correlated with markers of UPR, e.g. p-eIF2α (fig. 3C) indicating that JNK activity is associated with palmitate and glucose-induced ER stress.

**Figure 6:**
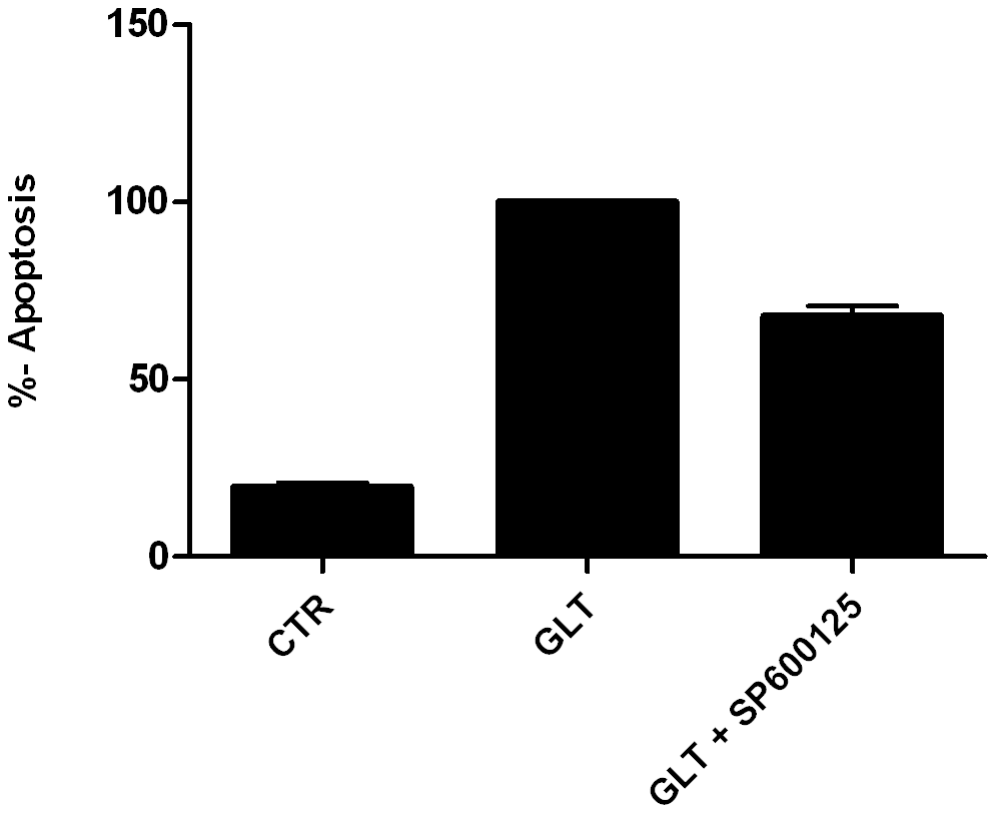
JNK inhibition by SP600125 inhibits palmitate and high glucose-induced apoptosis. Non-sense shRNA expressing INS1 cells were exposed to vehicle (CTR), 0.5 mM palmitate and 25 mM glucose (GLT) or GLT with 20 µM SP600125 for 24 h. Cells were pre-incubated with 20 µM SP600125 2 h prior to addition of GLT. Apoptosis was measured as the relative levels of cytoplasmic nucleosomes compared to CTR using the Cell Death detection ELISA Assay kit. Data from three independent experiments are shown as means+SEM.

We (fig. 6) and others have shown that blocking total JNK activity with the synthetic ATP-competetive JNK kinase inhibitor SP600125 *in vitro* ameliorates palmitate and/or high glucose-induced INS-1E and MIN-6 cell apoptosis [9], [21], [25]. However, the SP600125 inhibitor is neither JNK protein nor JNK subtype specific [35]. Here we show for the first time that JNK1 protects against palmitate and high glucose-induced INS1 apoptosis associated with modulation of mitochondrial and ER stress proteins. JNK2 knockdown did not affect palmitate and high glucose-induced apoptosis or any of the investigated ER stress markers, but increased *Puma* mRNA expression. It is possible that individual JNK proteins interact to mediate signal transduction, and thus removal of JNK1 may hinder protective signal transduction and β-cell survival in response to palmitate and high glucose, explaining why JNK2 knockdown *per se* did not protect against INS1 cell apoptosis or ER stress. It is well known that there is regulatory crosstalk between the JNK isoforms [36], and the balance between the JNK isoform expression deciding total JNK activity may be a critical determinant of β-cell survival [37]. Furthermore, individual JNK isoform activity may depend on context-dependent stimuli [38]. In this study analysis of subtype specific JNK activity was hampered by the unavailability of suitable specific anti-phospho JNK subtype antibodies, and as the JNK subtypes have differential binding affinities for target substrates, pull-down of the individual JNK subtype followed by kinase activity assay may not reflect the accurate JNK subtype activity.

JNK1, 2 or 3 knockdown did neither affected the PERK-pathway (fig. 3D, E, F), nor *ATF3* mRNA expression (fig. 3G–H). The role of ATF3 in β-cell death is controversial. Islets isolated from ATF3 knockout (KO) mice are partially protected against cytokine and NO-mediated apoptosis, and ATF3 is expressed in the islets of type 1 and type 2 diabetic patients and in the islets of non-obese diabetic mice, indicating a proapoptotic role of ATF3 during stress conditions [13]. On the other hand, ATF3 KO mouse islets exposed to high concentrations of glucose have decreased expression of *ins1* and *ins2* steady-state mRNA and insulin content and diminished glucose stimulated insulin secretion (GSIS) compared to wildtype mouse islets suggesting a beneficial role of ATF3 on β-cell function [39], although GSIS in ATF3 KO mouse islets has also been reported to be normal [40]. However, we observed reduced basal *ins1* and *ins2* mRNA expression (fig. 5E–F) in JNK1 shRNA expressing INS1 cells indicating that the insulin genes are regulated by ATF3 independent pathways. JNK1 knockdown INS1 cells also displayed decreased total basal insulin content (fig. 5G). Islets isolated from *JNK1^−/−^* mice or from wildtype mice treated with the peptide inhibitor of JNK, D-JNKi are protected from the inhibitory effect of palmitate on high glucose-stimulated insulin gene transcription without affecting insulin content [41]. Our data thus support a possible role of JNK1 in basal insulin transcription; however this needs further clarification.

Deletion of the ER stress marker CHOP decreases β-cell oxidative stress in several mouse models of diabetes [10]. Oxidative stress activates JNK and thereby contributes to β-cell apoptosis [26], [42]. Blocking glucolipotoxocity-induced ROS formation from mitochondria in β-cells decreases ER stress and reduces JNK activation [25]. Only JNK1 shRNA significantly increased CHOP mRNA and protein expression after palmitate and high glucose exposure compared to non-sense, JNK2 or JNK3 shRNA. CHOP transcription depends on the ATF4, AP-1 or Foxo1transcription factors [23], [43]. We did not observe upregulation of *ATF4* mRNA in response to palmitate and high glucose in JNK1 shRNA expressing INS1 cells pointing towards an AP-1 or Foxo1-dependent regulation of *CHOP* mRNA expression although this needs to be formally shown. JNK1 knockdown also increased ROS formation possibly associated with CHOP-dependent mechanism. This indicates that JNK1 abolishes the contribution of CHOP expression and ROS formation in response to β-cell glucolipotoxicity and that JNK1 is an important modulator of mitochondria to ER stress cross-talk.

JNK regulates the mitochondrial intrinsic death pathway through transcriptional and posttranslational modifications of Bcl-2 family-proteins [44], [45]. Therefore, we investigated the effects of JNK subtype shRNAs on the BH3-only “sensitizer” DP5 and “activator” Puma, two candidate Bcl-2 family proteins recently shown to be upregulated and to mediate palmitate-induced β-cell apoptosis by linking ER stress to the intrinsic death pathway [27]. Only JNK3 shRNA increased *DP5* mRNA expression when compared to non-sense shRNA. *Puma* mRNA expression was increased by JNK1, 2 or 3 shRNA expressing INS1 cells exposed to palmitate and high glucose for 12 h. At 16 h only JNK1 knockdown increased *Puma* expression. JNK-activated Jun and PERK-induced ATF3 regulates *DP5* expression, whereas *Puma* expression is JNK independent but ATF3-dependent by regulation of TRB3 and FoxO3a [27]. As we did not detect increased *ATF-3* mRNA expression in any of our JNK knockdown INS1 cell lines exposed to palmitate and high glucose *Puma* mRNA expression is likely upregulated by ATF-3 independent mechanisms in the individual JNK1, 2 or 3 knockout INS1 cell lines: however this needs further investigations.

Previous studies using JNK interference in cytokine-induced β-cell death have shown differential biological functions of the JNK subtypes. Abdelli et al. [46] found JNK3 to have antiapoptotic properties in *cytokine-*induced β-cell apoptosis whereas JNK1 and JNK2 were reported to serve proapoptotic functions in response to cytokines. Here we show that JNK1 exerts an antiapoptotic function in palmitate and high glucose-induced β-cell death. JNK2 knockdown did not influence palmitate and high glucose induced β-cell apoptosis and JNK3 knockdown only increased cleaved caspase 9 and 3 but not apoptosis. The difference between our and Abdellis data supports the idea that the JNK subtypes exhibit differential roles depending on stimuli. We suggest that palmitate and high glucose induce oxidative stress, ROS and ER stress associated with increased expression of the proapoptotic Bcl-2 proteins DP-5 and Puma. In summary, we propose that JNK1 protects against palmitate and high glucose-induced β-cell apoptosis, CHOP and *Puma* expression, and ROS formation.
